# A Secure and Efficient Digital-Data-Sharing System for Cloud Environments

**DOI:** 10.3390/s19122817

**Published:** 2019-06-24

**Authors:** Zhen-Yu Wu

**Affiliations:** Department of Information Management, National Penghu University of Science and Technology, Penghu 880, Taiwan; zywu@gms.npu.edu.tw; Tel.: +886-06-926-4115 (ext. 5211 or 5422)

**Keywords:** Education Cloud, confidentiality, privacy exposure, access control, digital sharing

## Abstract

“Education Cloud” is a cloud-computing application used in educational contexts to facilitate the use of comprehensive digital technologies and establish data-based learning environments. The immense amount of digital resources, data, and teaching materials involved in these environments must be stored in robust data-access systems. These systems must be equipped with effective security mechanisms to guarantee confidentiality and ensure the integrity of the cloud-computing environment. To minimize the potential risk of privacy exposure, digital sharing service providers must encrypt their digital resources, data, and teaching materials, and digital-resource owners must have complete control over what data or materials they share. In addition, the data in these systems must be accessible to e-learners. In other words, data-access systems should not only encrypt data, but also provide access control mechanisms by which users may access the data. In cloud environments, digital sharing systems no longer target single users, and the access control by numerous users may overload a system and increase management burden and complexity. This study addressed these challenges to create a system that preserves the benefits of combining digital sharing systems and cloud computing. A cloud-based and learner-centered access control mechanism suitable for multi-user digital sharing was developed. The proposed mechanism resolves the problems concerning multi-user access requests in cloud environments and dynamic updating in digital-sharing systems, thereby reducing the complexity of security management.

## 1. Introduction

As the name suggests, e-learning involves learning through digital media [1]. E-learning can be traced back to the Programmed Logic for Automatic Teaching Operations (PLATO) introduced by Prof. Patrick Suppes of Stanford University and Prof. Don Bitzer of the University of Illinois in 1960 [2]. The PLATO system was capable of teaching students reading and writing skills through courses involving computer-assisted instruction (CAI). E-learning has gradually evolved from standalone CAI to online learning platforms that cater to mass users [3]. For example, the online courses offered through the University of Phoenix’s online platform and through OpenCourseWare, operated by the Massachusetts Institution of Technology, provide learning opportunities for people all over the world through digital sharing.

Amidst advancements of network technologies, the prevalence of digital-multimedia-information use has accelerated. For example, application of digital multimedia information has become a mainstream in teaching. This approach not only overcomes the spatial and temporal constraints of learning but also contributes to more engaging, interactive, and immediate learning experiences, which motivate learners and stimulate their interests. These features support users’ comprehension of new content, and reinforce previously learned concepts [3,4,5,6]. E-learning achieved steady development worldwide after the National Institute of Standards and Technology (NIST) announced endorsement of cloud computing and the application of cloud computing in Internet applications in 2009 [7].

“Education Cloud” refers to the application of cloud computing [8] to establish data-based learning environments and use of comprehensive digital technologies in educational contexts. Related provisions are as follows:

(1) Create a learning environment centered on digital technology and data, provide teachers and students with an online education portal, and ensure that teachers are able to apply the various e-learning tools provided to them to teach their students.
(2) Establish a comprehensive online school network that offers wireless Internet and roaming mechanisms to facilitate teaching and learning at the school. Create a data application environment free of spatial constraints, and apply new technologies such as voice over Internet protocol to reduce administration cost and enhance interconnection efficiency
(3) Reduce digital gaps and create balanced digital environments to enable future students to develop new ways of learning through the novel teaching approach, thereby fostering their independent thinking and problem-solving abilities, which constitute the advantages of the new generations.
(4) Consolidate various cloud learning content and services and meet learner-centered resource demands; learn and identify suitable resources through the cloud to achieve cloud-based learning. Satisfy environmental demands for learning prosperity, autonomy, and convenience, thereby facilitating customized and autonomous learning.

In cloud-sharing environments, immense amounts of digital resources, data, and teaching materials must be stored in robust data-access systems. These systems must be equipped with effective security mechanisms to guarantee confidentiality and ensure the integrity of the cloud-computing environment [9,10,11]. To minimize the risk of privacy exposure, digital sharing service providers should provide encryptions for the digital resources, data, and teaching materials. The data-access systems must also offer editors and authors control over what data or materials they share. In addition, the data in these systems must be accessible to e-learners. In other words, data-access systems should allow conventional service providers to encrypt their data and should also provide control mechanisms by which users may access the data [12].

Access control is a form of access protection for data systems that prevents unauthorized users from corrupting, deleting, or modifying data. Specifically, it is a protection mechanism that governs what items are accessible to system users and the extent of the items’ accessibility. Therefore, access control is a key component in the field of network communication and data security. When a user wants to retrieve a document stored in a data system, the user’s identity is authenticated by a username and password to verify that the user possesses the necessary authorization to add, delete, modify, or access specific documents [13].

Cloud-based digital sharing systems are formatted to enable use by numerous users; however, provision of simultaneous access control to numerous users may overload a system’s computational capacity or increase the requirement for physical management of the system [12,14]. Authorized users and users requesting authorization may access the system from different channels. Generally, demands from existing users and new user requests are immense and unpredictable. If all users are able to manage their subscriber base directly, security management (management of encryption and decryption keys and certifications) will be complicated by the excessive number of users [15].

By directly storing digital resources in the cloud, authorized users can manage these resources from any location and at any time without depending on an online administrator to approve their request. In other words, the accessibility and usability of digital sharing systems are unrestricted. However, following the continual additions and modifications of content in digital sharing systems, the digital information stored in the cloud may originate from various cities and counties, and authorized users may submit requests to the cloud server at any time to retrieve the latest resources or teaching materials. Therefore, the dynamic update function of digital sharing systems must be implemented in the provision of cloud services.

Although previous studies have proposed various encryption techniques to prevent the unauthorized data access, most of these techniques are targeted at single-user systems. In cloud environments, digital sharing systems do not target single users; rather, they provide secure and efficient access control mechanisms that allocate different permissions to different users. To overcome the challenges of non-single-user applications and to harness the many benefits of combining digital sharing systems with cloud computing, we developed a cloud-based and learner-centered access control mechanism suitable for multi-user digital sharing. The mechanism resolves the problems related to multi-user access requests in cloud environments and dynamic updating in digital sharing systems, thereby reducing the complexity of security management.

The rest of this paper is organized as follows. Section 2 offers a review of related works. Section 3 proposes the digital-data-sharing system. Section 4 demonstrates the security of the proposed system and evaluates system performance. Finally, Section 5 concludes this paper.

## 2. Related Works

### 2.1. Access Control Mechanisms

Access control has been extensively studied, and scholars have proposed a variety of access control mechanisms, such as access control matrices, access control lists, capability lists, and role-based access control (RBAC). Access control matrices are the simplest of these mechanisms to use in management of system resource-access [16]. When a user submits a request to access system resources, the system uses the user’s position relative to the requested object in an access control matrix to determine the legitimacy of the request.

The concepts behind access control lists and capability lists are similar. Both mechanisms compile authorization logs into lists. In an access control list, authorization logs are compiled into columns in the access control matrix, where system resources constitute the base matrix and users are represented in a linked list. In a capability list, permission logs are compiled into rows in the access control matrix, where users constitute the base matrix, and system resources are represented in a linked list. Access control lists facilitate the management of system resource requests. However, searching for a specific user in an access control list is time intensive. By comparison, the advantages of capability lists correspond with the disadvantages of access control lists.

In addition to the aforementioned access control mechanisms, several access control models have been proposed. For example, task-based access controls (TBACs) authenticate access requests and periods based on task requirements [17], temporal RBACs (TRBAC) authenticate role permissions based on the changes in time intervals [18], rule set-based access controls authenticate permissions based on the system’s security strategy [19], and spatial RBAC (SRBAC) authenticates role permissions based on changes in spatial locations [20]. These access control models can be employed independently or combined with other control models. For instance, TBACs can be used simultaneously with RBACs in access systems [17], or a hybrid RBAC can be adopted as the access control mechanism in large and complex organizations [21]. Furthermore, access control mechanisms can be incorporated into other system structures, such as RBAC online-payment systems [22].

At present, commonly applied access controls can be categorized into three types: discretionary access control (DAC), mandatory access control (MAC), and RBAC.

In DAC mechanisms, access is granted based on user identity and the specific action related to the access request. Users are able to manage the access permission of the objects they own without intervention from system administrators. In sum, DAC enable the transfer of object authorities and is suitable for developing environments for data sharing and autonomous application of authority [23].

Although DAC models provide flexible access control mechanisms, they cannot guarantee the integrity of data after authorization [24]. For example, users that are authorized to access a particular document may download the document onto a storage device and then transfer the document to others without the authorization of the owner. Thus, in DAC systems, document owners are unable to track the authorized users’ transfer of their documents, and document receivers cannot determine whether the rights of the document belong to the document provider or whether the document was merely transferred from another source.

MACs prohibit users from freely allocating access permissions, and permission allocation rights belong exclusively to a system administrator [25]. MACs assign security levels and category labels to all subjects and objects in a system. When a user requests access to an object, the system compares the labels of the user and the object. If the users' access permissions correspond with or exceed the confidentiality level of the object, the request of the user is authorized; otherwise, the request is rejected [24]. For example, assume the system sets the security clearance of User A at 2 and that of User B at 4, and that the security levels of Documents A, B, and C are 1, 3, and 6, respectively. User A is able to view Document A, which is categorized under a security level lower than his or her clearance, whereas User B is authorized to view Documents A and B. The security level of Document C is higher than the clearance of both users; therefore, they cannot access Document C. The application of MAC is more complicated than that of DAC, making MAC-based systems suitable for environments with stringent security requirements, such as national defense departments. 

The RBAC was introduced by Sandhu et al. in 1996 [13] It was subsequently incorporated and standardized by the NIST in 2011, and was renamed as NIST RBAC [26]. RBACs create “role” elements between the “user” and “access permission” elements in a system, enabling users to access documents through the “role” element.

As awareness of digital data confidentiality rose in 2004, Chen et al. proposed an access control mechanism that combined encryption and key management, and applied the mechanism in a mobile agent environment [27,28,29]. Before a mobile agent is approved for work on the Internet, the transmitting host decides which hosts will be visited by and what data is accessible to the agent. In addition, the owner of the mobile agent must first determine pathways and access strategy. The owner of the agent then encrypts his or her confidential files with separate keys using a symmetric encryption system, such as the Advanced Encryption Standard (AES), Data Encryption Standard (DES), or International Data Encryption Algorithm [30]. Finally, various access permissions are established based on the access control strategy, and a hierarchical structure is created based on the level of the access permissions. The owner of the agent provides a superkey to each host and publishes specific public parameters of the agent. The hosts then use their superkeys to access data from hosts with security levels below their clearance.

Thereafter, access mechanisms based on an elliptic curve, bilinear pairing, and ID authentication, and mechanisms with migration and time constraints were sequentially introduced [31,32,33,34]. Cloud environments matured by 2012 and Liu et al. proposed a dynamic access framework that achieved accurate access control of cloud data and logs in a multi-user setup. The framework was incorporated into a medical environment to maximize patients’ control of their medical records. The system ensured privacy by only granting access to doctors, pharmacists, nurses, and researchers [14,15,35].

Recently, context-aware access control (CAAC) models have been developed, extending the basic RBAC authorization model, which determine whether users’ requests to limit the access permissions for privacy data and information based on the dynamically changing contextual conditions, such as related resources, environments, user properties, software services [36,37]. For example, Schefer-Wenzl and Strembeck proposed the fuzzy model with an ontology-based approach that captures contextual conditions for mobile business processes [38]. Hosseinzadeh et al. used ontological techniques and Web Ontology Language (OWL) of modeling context-aware role-based access control scheme for smart spaces [39]. Trnka and Cerny proposed a CAAC scheme based on using security levels, which are granted to user based on his/her context [40]. Colombo and Ferrari proposed a roadmap to enhance the data protection functionalities of NoSQL datastore and then design a CAAC mechanism for MongoDB [41,42]. Kayes et al. developed several CAAC systems by considering a wide variety of contextual conditions, the relationship context information utilizing the process of inferring implicit knowledge, and the purpose-oriented situation information based on the currently available context information [43,44,45].

### 2.2. Lagrange Interpolation Polynomial

Following, is a brief introduction to Lagrange interpolation polynomial [30], which we have adopted for encryption and decryption processes. In numerical analysis or other applications, many practical problems are represented through functions to express intrinsic relationships or regularity. However, the precise relationship between variable *x* and variable *y* of many functions are extremely complex, and cannot be determined through experiments. The method of Lagrange interpolation enables us to obtain a polynomial which passes through a finite set of points in the x-y plane. The polynomial obtained by this method is called the Lagrange polynomial. Mathematically, the Lagrange interpolation polynomial can obtain a polynomial function, which passes through known points of a two-dimensional plane. For example, in a x-y plane, given *n* + 1 are known points, (*x*_0_, *y*_0_), (*x*_1_, *y*_1_), …, (*x_n_*, *y_n_*). The method of Lagrange interpolation provides a formula for constructing a unique polynomial of degree *n* which passes through these *n* + 1 points. Among them, the Lagrange fundamental polynomial, or interpolation basis function is expressed as follows:

(1) $${𝓁}_{j}\left(x\right)=\overset{n}{\underset{i=0,i\neq j}{\prod }}\frac{x-x_{i}}{x_{j}-x_{i}}=\left(\frac{x-x_{0}}{x_{j}-x_{0}}\right)...\left(\frac{x-x_{j-1}}{x_{j}-x_{j-1}}\right)\;\left(\frac{x-x_{j+1}}{x_{j}-x_{j+1}}\right)\;...\left(\frac{x-x_{n}}{x_{j}-x_{n}}\right)\;,\;1\leq j\leq n$$

The specific point of *l_j_*(*x*) is the derived value 1 from *x_j_*. Values from other points *x_i_* (*i ≠ j*) equals 0, the expression of which is as follows: ${𝓁}_{j}\left(x\right)=\{\begin{array}{c} \begin{array}{cc} 0, & i\neq j \end{array}\\ \begin{array}{cc} 1, & i=j \end{array} \end{array}$. The Lagrange polynomial is $L\left(x\right)=\overset{n}{\underset{j=0}{\sum }}y_{j}{𝓁}_{j}\left(x\right)$.

That is the unique polynomial of degree *n* which passes through the points (*x*_0_, *y*_0_), (*x*_1_, *y*_1_), …, (*x_n_*, *y_n_*). For example, the binomial that passes through (4, 1), (5, 5), and (6, 10) when expressed in Lagrange basic polynomial is as follows

$${𝓁}_{1}\left(x\right)=\left(\frac{x-5}{4-5}\right)\left(\frac{x-6}{4-6}\right),\;{𝓁}_{2}\left(x\right)=\left(\frac{x-4}{5-4}\right)\left(\frac{x-6}{5-6}\right),\;{𝓁}_{3}\left(x\right)=\left(\frac{x-4}{6-4}\right)\left(\frac{x-5}{6-5}\right).$$

By applying Lagrange interpolation polynomial, a single polynomial *L*(*x*) can be obtained as expressed below:

$$\begin{array}{cc} L\left(x\right) & =f\left(4\right)l\left(1\right)+f\left(5\right)l\left(2\right)+f\left(6\right)l\left(3\right)\\ & =1\times \left(\frac{x-5}{4-5}\right)\left(\frac{x-6}{4-6}\right)+5\times \left(\frac{x-4}{5-4}\right)\left(\frac{x-6}{5-6}\right)+10\times \left(\frac{x-4}{6-4}\right)\left(\frac{x-5}{6-5}\right)\;\\ & =\frac{1}{2}x^{2}-\frac{1}{2}x-5 \end{array}$$

It can be inferred that *f*(4) = 1, *f*(5) = 5, *f*(6) = 10. By applying this formula, predicted values can be derived, for example: to derive *f*(18), substitute *x* = 18 in *L*(*x*), and *L*(18) = *f*(18) = 148 is derived.

## 3. The Proposed Mechanism

**Figure 1:**
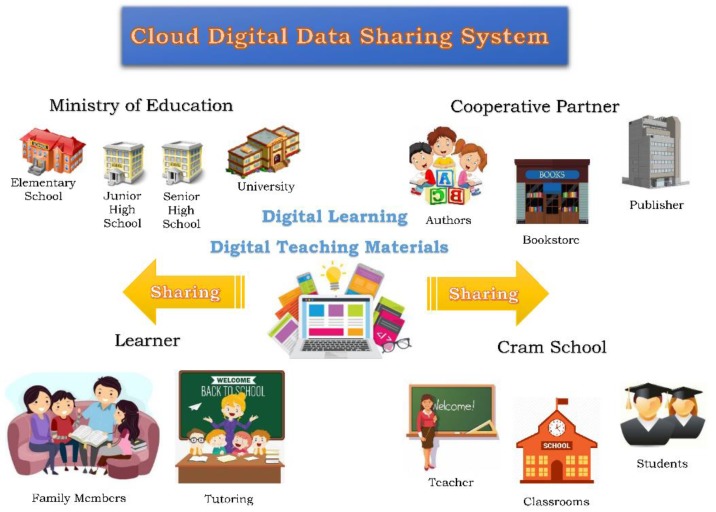
Access diagram for the cloud-based digital sharing system.

The foremost challenges when creating digital-sharing systems for cloud environments are managing large user bases and complex access relationships. Ensuring the confidentiality and integrity of users’ cloud data represents an additional concern. In response to these challenges, we developed a dynamic multi-user access mechanism that can accurately access and control the digital resources and teaching materials stored in the cloud, as shown in Figure 1. The proposed systems indexes Lagrange interpolating polynomials to provide different users maximum control over their data and logs. The system also comprises an encryption technique to protect users’ privacy, with unique keys generated by Central Authority that can be freely used to share their digital data. The steps involved in developing the proposed access control system are explained in the following section. The definitions of the symbols used in the creation of the proposed system are tabulated in Table 1.

### 3.1. Create a System User

In this study, we adopt the access relationships of a partially ordered set. A Central Authority (CA) (or a multiple number of CAs that are distributed by a single CA) builds the partially ordered set, which is a pair (*S*, ≼), where ≼ is a reflexive, antisymmetric, transitive binary relation in set *S*. In this paper, users are divided into disjoint sets labeled *S_i_*, where *S_i_* is a subset that corresponds with security classes, and each class is assigned clearance to access specific files. Therefore, the decryption key with permission to obtain the encrypted file can be expressed as *S_i_* = {*u*: *u* is the file id that *S_i_* is permitted to access} for *i* = 1, 2, …, *n*, where *n* ∈ *Ν* and ‘≼’ is a binary partial order relation over the set *S* = {*S*_1_, *S*_2_, …, *S_n_*}. For the set (*S*, ≼), *S_j_* ≼ *S_i_* (*i*, *j* ∈ *Ν*), indicating that a user in security class *S_i_* can read or store data held by a user in security class *S_j_*, but the opposite is not allowed. Each class possesses its own cryptographic key; thus, if *S_j_* = {1, 2}, *S_i_*_­_ = {1, 2, 3}, {1, 2} ≼ {1, 2, 3}, then *S_j_* ≼ *S_i_*. In *S_j_* ≼ *S_i_*, *S_i_* corresponds with the security class required to obtain the decryption key for *S_j_* to retrieve *file*_1_ and *file*_2_.

The system may be accessed by users of a variety of identities, such as teaching material authors, partner vendors, authorities affiliated with the Ministry of Education, class teachers, students, and students’ parents. In the proposed system, the security class of each user is expressed as *S_i_*, and each user possesses a superkey (*H_i_*), where *i* = 1, 2, ..., *n*. The CA creates a framework for these users. The system structure comprises *n* users in two sets, *S* = {*S*_1_, *S*_2_, ..., *S_n_*} and *H* = {*H*_1_, *H*_2_, ..., *H_n_*}, which can be expressed in Table 2: 

### 3.2. Establish an Associative Array and Function for System Users and Data Files

The digital-data-sharing system proposed in this study was developed specifically for teaching-material-related use. The proposed system stores data provided by publishers (partner vendors), the Ministry of Education, teaching material authors, and teachers. The system applies encryption keys to the data uploaded by the various users to generate encrypted files, which are then stored in the cloud server. The CA builds a structure in which *m* files form a set *file* = {*file*_1_, *file*_2_, ..., *file_m_*}. Additionally, the CA creates decryption keys corresponding to *file_u_*, where *u* = 1, 2, ..., *m*, protecting the encrypted files from random access. The decryption keys are expressed as *DK_u_*, where *u* = 1, 2, ..., *m.* The relationship between the files and keys can be shown in Table 3.

The following adjacency matrix illustrates the access relationships. Assume the system structure comprises six security classes and four files; {security classes} × {files} data may be arranged in a two-dimensional array as follows:

$$\begin{array}{cccc} & & \begin{array}{cccc} file_{1} & file_{2} & file_{3} & file_{4} \end{array} & \\ \begin{array}{c} S_{1}\\ S_{2}\\ S_{3}\\ S_{4}\\ S_{5}\\ S_{6} \end{array} & [ & \begin{array}{ccccccccc} 1 & & & 1 & & 1 & & & 1\\ 1 & & & 1 & & 1 & & & 0\\ 0 & & & 1 & & 1 & & & 1\\ 1 & & & 1 & & 0 & & & 0\\ 0 & & & 1 & & 1 & & & 0\\ 0 & & & 0 & & 1 & & & 1 \end{array} & ] \end{array}$$

We define the indicate function as *I*(*x*, *y*). This function expresses that user *i* is permitted to obtain *file_u_* using *DK_u_*. Variable *x* represents user’s id *i*, and the variable *y* represents *file*’s id *u*. User *i* uses his or her secret superkey *H_i_* to access row *i*. According to the construction, row *i* contains the set of *file_u_* that user *i* is authorized to access. For example, *I*(3, 2) = 1 because user 3 is authorized to access *file*_2_. *I*(6, 1) = 0 because user 6 is not authorized to access *file*_1_.

(2) $$I\left(x,y\right)=\{\begin{array}{l} \begin{array}{cc} 1 & ,\;\mathrm{if}\;\mathrm{user}\;x\;\mathrm{has}\;\mathrm{access}\;\mathrm{to}\;\mathrm{file}\;y \end{array}\\ \begin{array}{cc} \;0 & ,\;\mathrm{otherwise} \end{array} \end{array}$$

**Figure 2:**
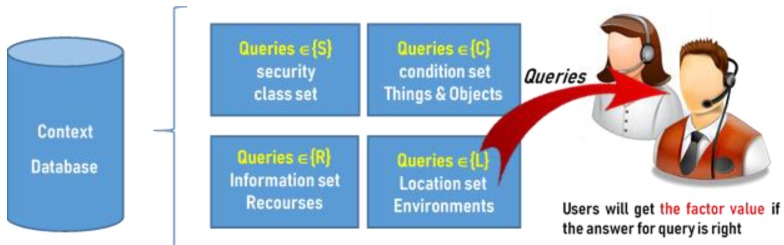
Context database and query set for the proposed digital sharing system.

For a flexible specification of access control policy, we combine the dynamically changing context by using the particular context database. It is mainly formed with 3w queries (who, what, and where), i.e., the sets of questions for judgement the specific people (1) whether he/she comes from the security class {*S*}; (2) what conditions that he/she needs to handle (specific information and resources to be obtained); and (3) what locations that he/she may exist (the determination of environments). Figure 2 shows the detailed structure of the database. Here, we require that before people, who have authority (i.e., *I*(*x*, *y*) = 1), access the file, he/she must firstly pass the queries from the context database.

Each query may equally come from different sets and the requested users need to send the right answers for the response queries, making the sum of value of identification factors be greater than, or equal to, one, i.e., $\text{\{}factor_{Q_{n}}|\;0\leq factor_{Q_{n}}\leq 1,\;\mathrm{for}\;i=1,2,\ldots ,n\}$, where $factor_{Q_{1}}+factor_{Q_{2}}+\ldots +factor_{Q_{n}}\geq 1$. The system can determine that the user has indeed been authorized and owned the right to get secure key and access the file contents by the sum of the factor value reaching to one.

### 3.3. Establish the Correlation Functions to Derive Keys of System Users

To accurately derive *DK*s to access the desired file, numerous auxiliary polynomials and functions are stored in the system to assist in the processing of access control. First, the authenticity of users’ keys must be defined. Therefore, the indicator function is defined as $I_{\left\{H_{1},...,H_{n}\right\}}\left(x\right)=\{\begin{array}{c} \begin{array}{lll} 1 & ,if & x\in \left\{H_{1},...,H_{n}\right\} \end{array}\\ \begin{array}{ccc} 0 & ,\;o.w. & \end{array} \end{array}$, where an output result of 1 represents an authentic key. For other outputs, the key is rejected.

Second, the clearance of the user must be determined for file access to be granted. Therefore, the function is defined as *J_i_* = {*u*: 1 ≤ *u* ≤ *m*, *u* is the file id that *S_i_* has permission to access} (assuming that the system contains *n* users and *m* files for access). The preceding two auxiliary functions form the function $I_{J_{i}}\left(y\right)$, which expresses that the user’s *S_i_* is authorized to access the *DK*. The function can be expressed as follows:

(3) $$I_{J_{i}}\left(y\right)=\{\begin{array}{c} \begin{array}{ccc} 1 & ,if & y\in J_{i} \end{array}\\ \begin{array}{ccc} 0 & ,o.w. & \end{array} \end{array}$$

Third, several correlation functions can be generated by applying the Lagrange interpolation polynomial as below steps:

Step 1: the CA establishes a unique superkey *H_i_*, where *i* = 1, 2, ..., *n*, for user *i* in the *S* = {*S*_1_, *S*_2_, ..., *S_n_*} set. The *H_i_* is confidential to the user *i*.
Step 2: the CA manages the *H_i_* of all users and establishes an indicator function to authenticate the superkey: $I_{\left\{H_{1},...,H_{n}\right\}}\left(x\right)=\{\begin{array}{c} \begin{array}{lll} 1 & ,if & x\in \left\{H_{1},...,H_{n}\right\} \end{array}\\ \begin{array}{lll} 0 & ,o.w. & \end{array} \end{array}$.$\;I_{\left\{H_{1},\;\;...,H_{n}\right\}}\left(x\right)$ indicates that the indicator function of set *H* = {*H*_1_, *H_2_*, ..., *H*_n_}. $I_{\left\{H_{1},...,H_{n}\right\}}\left(x\right)$ is used to verify the authenticity of *H_i_*.
Step 3: the CA establishes the function *A_i_*(*x*) applying Lagrange interpolation polynomial for user *i*, where $A_{i}\left(x\right)=\left\{\overset{n}{\underset{\begin{array}{c} k=1\\ k\neq i \end{array}}{\prod }}\frac{\left(x-H_{k}\right)}{\left(H_{i}-H_{k}\right)}\right\}\times I_{\left\{H_{1},...,H_{n}\right\}}\left(x\right)$, for *i* = 1, 2, …, *n*, *x* ∈ *R*.
Step 4: the CA selects nonrepeated random integers {*DK_1_*, *DK_2_*, …, *DK_m_*} (supposing *m* confidential files exist) as the decryption key for encrypting and decrypting confidential files. The CA maintains the confidentiality of the *DK_u_* and publishes the public parameter *u*.
Step 5: the CA defines *J_i_* = {*u*: 1 ≤ *u* ≤ *m*, *u* is the file id that *S_i_* has permission to access} when *n* users exist for *i* = 1, 2, ..., *n* and *m* files for *u* = 1, 2, ..., *m*. *J_i_* is the set of *file* id’s that user *i* is authorized to visit.
Step 6: the CA defines $I_{J_{i}}\left(y\right)=\{\begin{array}{c} \begin{array}{ccc} 1 & ,if & y\in J_{i} \end{array}\\ \begin{array}{ccc} 0 & ,o.w. & \end{array} \end{array}$. This indication function expresses that user *i* is authorized to access the *DK_u_*. The function *B_i_*(*y*) is established by applying Lagrange interpolation polynomial for each user *i*. Let $B_{i}\left(y\right)=\left\{\underset{u\in J_{i}}{\sum }DK_{u}\left[\overset{m}{\underset{\begin{array}{c} t=1\\ t\neq u \end{array}}{\prod }}\frac{\left(y-t\right)}{\left(u-t\right)}\right]\right\}\times I_{J_{i}}\left(y\right)$, *y* ∈ *R*.
Step 7: the CA establishes the key-deriving function $G\left(x,y\right)=\overset{n}{\underset{i=1}{\sum }}A_{i}\left(x\right)B_{i}\left(y\right)$, *x* ∈ *R*, *y* ∈ *R*. That is, *G*(*x*, *y*) = *A_1_*(*x*)*B_1_*(*y*) + *A_2_*(*x*)*B_2_*(*y*) + … + *A_n_*(*x*)*B_n_*(*y*) for (*x*, *y*) ∈ *R* × *R*, and the CA declares it publicly.

Continually, user *i* can incorporate the owned superkey *H_i_* and the file id *u* to *G*(*x*, *y*) for deriving the *DK_u_*, which is then used to decrypt *file_u_*. The derivation process is described in the following steps:

Step 1: user *i* incorporates the superkey *H_i_* into $I_{\left\{H_{1},...,H_{n}\right\}}\left(x\right)=\{\begin{array}{l} \begin{array}{ccc} 1 & ,if & x\in \left\{H_{1},...,H_{n}\right\} \end{array}\\ \begin{array}{ccc} 0 & ,o.w. & \end{array} \end{array}$. If user *i*’s superkey *H_i_* is present in the authentication list established by the CA, then $H_{i}\in \left\{H_{1},...,H_{n}\right\}$ and $I_{\left\{H_{1},...,H_{n}\right\}}\left(H_{i}\right)=1$. If user *i*’s superkey *H_i_* is not present in the authentication list, then $I_{\left\{H_{1},...,H_{n}\right\}}\left(H_{i}\right)=0$.
Step 2: user *i* incorporates the superkey *H_i_* into $A_{i}\left(x\right)=\left\{\overset{n}{\underset{\begin{array}{c} k=1\\ k\neq i \end{array}}{\prod }}\frac{\left(x-H_{k}\right)}{\left(H_{i}-H_{k}\right)}\right\}\times I_{\left\{H_{1},...,H_{n}\right\}}\left(x\right)$. If user *i* uses his or her superkey *H_i_* and the superkey *H_i_* is present in the authentication list established by the CA, then $I_{\left\{H_{1},...,H_{n}\right\}}\left(x\right)=1$ can be incorporated for calculation. In this instance, *A_i_*(*H_i_*) = 1 and *A_i_*(*H_k_*) = 0 for *k* ≠ *i*.
Step 3: user *i* incorporates the id *u* of the desired *file_u_* into $I_{J_{i}}\left(y\right)=\{\begin{array}{c} \begin{array}{ccc} 1 & ,if & y\in J_{i} \end{array}\\ \begin{array}{ccc} 0 & ,o.w. & \end{array} \end{array}$, where *J_i_* = {*u*: 1 ≤ *u* ≤ *m*; *u* is the file id that *S_i_* has permission to access }. If user *i* is authorized to access *DK_u_*, then *y* ∈ *J_i_* and $I_{J_{i}}\left(y\right)=1$.
Step 4: user *i* incorporates the id *u* of the desired *file_u_* into $B_{i}\left(y\right)=\left\{\underset{u\in J_{i}}{\sum }DK_{u}\left[\overset{m}{\underset{\begin{array}{c} t=1\\ t\neq u \end{array}}{\prod }}\frac{\left(y-t\right)}{\left(u-t\right)}\right]\right\}\times I_{J_{i}}\left(y\right)$. If user *i* is authorized to access *DK_u_*, then *B_i_*(*y*) = *DK_y_* if *y* ∈ *J_i_* or *B_i_*(*y*) = 0 if *y* ∉ *J_i_*.
Step 5: user *i* calculates $G\left(x,y\right)=\overset{n}{\underset{i=1}{\sum }}A_{i}\left(x\right)B_{i}\left(y\right)$. If *x* ∈ {*H_1_*, *H_2_*, …, *H_n_*} and *y* ∈ *J_x_*_._, then *G*(*x*, *y*) = *DK_y_*. In this instance, the user can derive the decryption key; otherwise, *G*(*x*, *y*) = 0.

### 3.4. Change the Access Permissions of Users

The system users’ membership system may be added or removed due to different events, or as time changes. Additionally, users’ access permissions may change, and data may be added, modified, or deleted according to different access requirement. In this study, we developed an approach to resolve management problems related to system access security without sacrificing computing power and storage space.

The proposed system calculates the public function *G*(*x*, *y*). The following goals may be achieved by updating the function and modifying the parameters: (1) add user; (2) remove user, and (3) update user permissions.

(4) $$G\left(x,y\right)=\overset{n}{\underset{i=1}{\sum }}A_{i}\left(x\right)B_{i}\left(y\right),x\in R,y\in R$$

Further decomposition of *G*(*x*, *y*) yields *A_1_*(*x*)*B_1_*(*y*) + *A_2_*(*x*)*B_2_*(*y*) + … + *A_n_*(*x*)*B_n_*(*y*) where (*x*, *y*) ∈ *R*×*R* and the subfunction *A_i_*(*x*) is correlated to the authentication of user data. The subfunction verifies whether *H_i_* is present in a legitimate list in the system and whether the user can acquire a personal key for authentication. Additionally, subfunction *B_i_*(*y*) is correlated to data authentication. These subfunctions verify whether users can acquire *DK_u_* to decrypt encrypted data files. *A_i_*(*x*) and *B_i_*(*y*) can be expressed as follows:

(5) $$A_{i}\left(x\right)=\left\{\overset{n}{\underset{\begin{array}{c} k=1\\ k\neq i \end{array}}{\prod }}\frac{\left(x-H_{k}\right)}{\left(H_{i}-H_{k}\right)}\right\}\times I_{\left\{H_{1},...,H_{n}\right\}}\left(x\right),\;\mathrm{for}\;i=\;1,\;2,\;\ldots ,\;n,\;x\text{∈}\;R$$

(6) $$B_{i}\left(y\right)=\left\{\underset{u\in J_{i}}{\sum }DK_{u}\left[\overset{m}{\underset{\begin{array}{c} t=1\\ t\neq u \end{array}}{\prod }}\frac{\left(y-t\right)}{\left(u-t\right)}\right]\right\}\times I_{J_{i}}\left(y\right),\;y\text{∈}\;R$$

(1) Add user: to add a user, the system merely update the indication functions $I_{\left\{H_{1},...,H_{n+1}\right\}}\left(x\right)$ and $I_{J_{v}}\left(y\right)$ and creates *A_v_*(*x*), *B_v_*(*y*), and *J_v_* for the new user *S_v_*, after which the data are updated to *G*(*x*, *y*). In other words, *G’*(*x*, *y*) = *G*(*x*, *y*) + *A_v_*(*x*)*B_v_*(*y*). Only simple additive operation is involved in the computation.
(2) Remove user: similar to the process for adding a user, the system removes the *A_v_*(*x*) and *B_v_*(*y*) parameters associated with member *S_v_* from *G*(*x*, *y*) to remove a user. Therefore, *G’*(*x*, *y*) = *G*(*x*, *y*) − *A_v_*(*x*)*B_v_*(*y*). A subtraction algorithm is used in the computation.
(3) Update user access permissions: when a system user wishes to modify their access permissions, the system redefines *J_i_ ’* = {*u*: 1 ≤ *u* ≤ *m*, *u* is the file id that *S_i_* has permission to access }, where *J_i_’* represents the updated *S_i_* permissions. *B_i_*(*y*) is then updated to *B_i_’*(*y*); that is, the function *J_i_* related to *B_i_*(*y*) is replaced by *J_i_’* to obtain *G’*(*x*, *y*) = *G*(*x*, *y*) − *A_i_*(*x*)*B_i_*(*y*) + *A_i_*(*x*)*B_i_’*(*y*), reflecting the new permissions for the user. Addition and subtraction algorithms are used in the computation.

#### 3.4.1. Adding a New Security Class

In case that *S_v_* is a new security to be inserted into the user hierarchy; CA executes the procedure below for inserting the new security class *S_v_*.

Step 1: CA distributes the secret parameter superkey *H_v_* to a new security class *S_v_*.
Step 2: CA establishes *A_v_*(*x*). *A_v_*(*x*) is identical to that of *A_i_*(*x*) except that *n* is replaced by *n* + 1, $A_{v}\left(x\right)=\overset{n+1}{\underset{\begin{array}{c} v=1\\ v\neq k \end{array}}{\prod }}\frac{x-H_{k}}{H_{v}-H_{k}}$. The index $I_{\left\{H_{1},...,H_{n+1}\right\}}\left(x\right)=\{\begin{array}{l} \begin{array}{ccc} 1 & ,if & x\in \left\{H_{1},...,H_{n+1}\right\} \end{array}\\ \begin{array}{ccc} 0 & ,o.w. & \end{array} \end{array}$ is updated.
Step 3: CA establishes the parameter *J_i_* = {*u*: 1 ≤ *u* ≤ *m*, *u* is the file ID of authorized *S_i_*} for *S_v_*
Step 4: CA establishes *B_v_*(*y*), where $B_{v}\left(y\right)=\left\{\underset{u\in J_{v}}{\sum }DK_{u}\left[\overset{m}{\underset{\begin{array}{c} t=1\\ t\neq u \end{array}}{\prod }}\frac{\left(y-t\right)}{\left(u-t\right)}\right]\right\}\times I_{J_{v}}\left(y\right)$.
Step 5: The index $I_{J_{v}}\left(y\right)=\{\begin{array}{l} \begin{array}{ccc} 1 & ,if & y\in J_{v} \end{array}\\ \begin{array}{ccc} 0 & ,o.w. & \end{array} \end{array}$ is updated.
Step 6: CA updates formula *G*(*x*, *y*) in the original scheme that the new formula appears *G’*(*x*, *y*) = *G*(*x*, *y*) + *A_v_*(*x*)*B_v_*(*y*)

In the above process to append a user, CA simply updates the indices $I_{\left\{H_{1},...,H_{n+1}\right\}}\left(x\right)$ and $I_{J_{v}}\left(y\right)$ and establishes *A_v_*(*x*), *B_v_*(*y*), *J_v_* for the new security class *S_v_*. The information is updated to formula *G*(*x*, *y*). Few costs are required for computing the new security class *S_v_*, and merely addition is required for updating the entire scheme.

#### 3.4.2. The Example of Adding a New Security Class

In this example, we assume that the digital-sharing mechanism contained the security classes *S*_1_ through *S*_6_ and the digital resources file_1_ to file_5_. A downstream bookstore joins the sharing mechanism as *S*_7_ and the owner receives authorization to access Junior High School Year 1 English, Senior High School Year 2 Physics, and Senior High School Year 3 Chemistry (Table 4).

First, the CA assigns the superkey *H*_7_ to the downstream bookstore and updates the indication functions to $I_{\left\{H_{1},...,H_{n+1}\right\}}\left(x\right)$ and $I_{J_{v}}\left(y\right)$ according to the digital-resource permissions of the bookstore. The CA defines *J*_7_ = {1, 3, 4} for *S*_7_ and creates the following equation:

$$A_{7}\left(x\right)=\left\{\frac{\left(x-H_{1}\right)\left(x-H_{2}\right)\left(x-H_{3}\right)\left(x-H_{4}\right)\left(x-H_{5}\right)\left(x-H_{6}\right)}{\left(H_{7}-H_{1}\right)\left(H_{7}-H_{2}\right)\left(H_{7}-H_{3}\right)\left(H_{7}-H_{4}\right)\left(H_{7}-H_{5}\right)\left(H_{7}-H_{6}\right)}\right\}\times I_{\left\{H_{1},...,H_{7}\right\}}\left(x\right)$$

$$\begin{array}{c} B_{7}\left(y\right)=\{DK_{1}\times \frac{\left(y-2\right)\left(y-3\right)\left(y-4\right)\left(y-5\right)}{\left(1-2\right)\left(1-3\right)\left(1-4\right)\left(1-5\right)}+DK_{3}\times \frac{\left(y-1\right)\left(y-2\right)\left(y-4\right)\left(y-5\right)}{\left(3-1\right)\left(3-2\right)\left(3-4\right)\left(3-5\right)}\\ +DK_{4}\times \frac{\left(y-1\right)\left(y-2\right)\left(y-3\right)\left(y-5\right)}{\left(4-1\right)\left(4-2\right)\left(4-3\right)\left(4-5\right)}\}\times I_{J_{7}}\left(y\right) \end{array}$$

Finally, all parameters are updated into a new formula *G’*(*x*, *y*) = *G*(*x*, *y*) + *A*_7_(*x*)*B*_7_(*y*)

#### 3.4.3. Removing an Existing Security Class

Assuming that an existing security class *S_v_* is to be removed from the digital-sharing mechanism, CA could precede the following Step 1 or Step 2.

Step 1: CA removes the relevant parameter *A_v_*(*x*)*B_v_*(*y*) in the security class *S_v_* from *G*(*x*, *y*). *G’*(*x*, *y*) = *G*(*x*, *y*) − *A_v_*(*x*)*B_v_*(*y*)
Step 2: *J_v_* is defined as the set of *file* ID’s, which the user *v* is authorized to visit. Instinctively, CA updates *J_v_* and deletes the authorization of the user: *J_v_’* = ϕ = empty set

#### 3.4.4. The Example of Removing an Existing Security Class

Assuming that *S_7_* downstream bookstore in the original scheme is no longer authorized, CA tends to remove *S_7_* from the scheme, as below (Table 5): 

CA could choose one of the following methods to remove *S_7_*; one is to update formula *G’*(*x*, *y*) = *G*(*x*, *y*) − *A_7_*(*x*)*B_7_*(*y*) to remove the relevant parameters in *S_7_* and the other is to update *J_7_’* = ϕ so that *S_7_* could not pass the authorization verification. 

#### 3.4.5. Updating a User Authorized

In the initial phase of the proposed scheme, CA would establish the access authority for the security class *S_i_*. When a user is updated by the system authorization, CA would proceed the following steps.

Step 1: CA resets *J_i_’* = {*u*: 1 ≤ *u* ≤ *m*, *u* is the file ID of authorized *S_i_*}. *J_i_’* presents the new authorization of *S_i_* after update. When the authorization to the digital-sharing mechanism is changed, CA would re-calculate the adjacency matrix to generate a new set *J_i_*.
Step 2: CA updates *B_i_*(*y*) to *B_i_’*(*y*), as *J_i_* is replaced by *J_i_’* and the information of *J_i_* is relevant with *B_i_*(*y*). Assuming that a new authorization of set *J_i_’* is given to user *i*, then *G’*(*x*, *y*) = *G*(*x*, *y*) − *A_i_*(*x*)*B_i_*(*y*) + *A_i_*(*x*)*B_i_’*(*y*).

According to the above steps, the establishment of *J_i_* could easily update the authorization of user *i* to access to digital data. When the user *i* does not present any authorization, *B_i_*(*y*) does not need to be updated, but just take *J_i_’* = ϕ = empty set.

#### 3.4.6. The Example of Updating a User Authorized

Assuming that *S_4_* student could access to *file_4_* Chemistry in the original scheme, but no longer could after the research project being changed, a new authorization allows to access to *file_2_* mathematics, as below (Table 6): 

CA updates *J_4_* = {4} to *J_4_’* = {2} and updates *B_4_’*(*y*). Then *G’*(*x*, *y*) = *G*(*x*, *y*) − *A_4_*(*x*)*B_4_*(*y*) + *A_4_*(*x*)*B_4_’*(*y*)

$$B_{4}\prime \left(y\right)=\left\{DK_{2}\times \frac{\left(y-1\right)\left(y-3\right)\left(y-4\right)\left(y-5\right)}{\left(2-1\right)\left(2-3\right)\left(2-4\right)\left(2-5\right)}\right\}\times I_{J_{4}}\left(y\right)$$

In this dynamic access section, the construction and updating of *G*(*x*, *y*) involve only simple arithmetic calculations. These can be done on a fly for a system consisting of millions of servers and millions of files. This scheme is easy to operate as the user *i* just enters a pair of valid (*H_i_*, *u*) to get the correct *DK_u_*. The system administrator calculates and updates *G*(*x*, *y*) in the background in real time. Every server follows exactly the same operational steps to retrieve the correct decryption key.

## 4. Analysis of Security

In this section, a security analysis is performed to examine whether the proposed scheme is secure in practical applications. The analysis focuses on four types of attack that may affect the system’s security.

### 4.1. Equation Attack

Equation attack: attackers attempt to use a public formula *G*(·) to crack polynomials using mathematical algorithms and obtain the *DK_u_*.

Equation attacks occur during updates of users’ permissions. When one user is being removed while the other users remain unchanged, attackers can subtract old public data *G*(·) with new public data *G’*(·), or *G’*(·) − *G*(·) = 0, to derive *DK_u_*. The mechanism designed in this study can withstand equation attacks. In Section 3, we propose the following three dynamic update methods:

1. Addition of a new security class: *G’*(*x*, *y*) = *G*(*x*, *y*) + *A_v_*(*x*)*B_v_*(*y*)
2. Deletion of a security class: *G’*(*x*, *y*) = *G*(*x*, *y*) − *A_v_*(*x*)*B_v_*(*y*)
3. Update of a user’s authorization G’(x, y) = G(x, y ) − A_i_(x)B_i_(y) + A_i_(x)B_i_’(y)

In all three dynamic update methods, the public parameters *G*(*x*, *y*) from before the update are subtracted from the updated public parameters *G’*(*x*, *y*). Therefore, attackers can only derive *A_v_*(*x*)*B_v_*(*y*) or *A_i_(x)B_i_(y) + A_i_(x)B_i_’(y)*. In the proposed methods, both *A_v_*(*x*) and *B_v_*(*y*) are polynomials created through Lagrange interpolation. Therefore, a multiplication algorithm must be applied to convert *A_v_*(*x*) and *B_v_*(*y*) into an (*n* − 1)(*m* − 1)^th^ order polynomial with two unknowns.

(7) $$A_{v}\left(x\right)=\left\{\overset{n}{\underset{\begin{array}{c} u=1\\ u\neq v \end{array}}{\prod }}\frac{\left(x-H_{u}\right)}{\left(H_{i}-H_{u}\right)}\right\}\times I_{\left\{H_{1},\ldots ,H_{n}\right\}}\left(x\right)=a_{0}+a_{1}x+\ldots +a_{n-1}x^{n-1}$$

(8) $$B_{v}\left(y\right)=\left\{\underset{j\in J_{v}}{\sum }DK_{u}\left[\overset{m}{\underset{\begin{array}{c} t=1\\ t\neq u \end{array}}{\prod }}\frac{\left(y-t\right)}{u-t}\right]\right\}\times I_{J_{v}}\left(y\right)=b_{0}+b_{1}y+\ldots +b_{m-1}y^{m-1}$$

*A_v_*(*x*)*B_v_*(*y*) = *a*_0_*b*_0_ + *a*_1_*b*_0_*x* + *a*_0_*b*_1_*y* + *a*_1_*b*_1_*xy* …+ *a*_*n*−1_*b*_*m*−1_*x*^*n*−1^*y*^*m*−1^ (9)

If the attacker incorporates *x* = 0 or *y* = 0 into the deduction, the returned polynomial messages of *A_v_*(*x*)*B_v_*(*y*) would comprise a string of unstructured data. Therefore, our methods are not vulnerable to compromising attacks.

### 4.2. External Attack

External attack: external users attempt to use public parameters to gain access. They attempt to obtain *DK_u_* or decrypt documents to acquire a digital resource stored in the cloud.

Digital teaching materials, data, and data sources acquired from the cloud can be sold at a low price, which not only infringes upon the authors' intellectual property rights, but also causes immense losses for publishers. For an unauthorized external user to access digital resources in the proposed digital sharing mechanism, the user must use public parameters to derive the decryption key and decrypt the files to acquire meaningful data.

The most critical known public parameter for external attackers is the public function *G*(*x*, *y*), because this function contains the *DK_u_*. Therefore, the equations based on this function must be protected. In the proposed method, each security class *S_i_* can be incorporated into private superkeys *H_i_* using the public function *G*(*x*, *y*) to derive the *DK_u_*. If an external attacker attempts to obtain the *DK_u_*, he or she must decrypt the Lagrange interpolating polynomials to obtain a secret key. For external attackers that can only obtain the public function *G*(*x*, *y*) and file id *u*, the large number of unknown variables hinders them from reverse deriving the *DK_u_* through mathematical computation. Therefore, attackers cannot unlawfully acquire digital teaching materials, data, or data sources through external attacks on the proposed system.

In the proposed method, the CA can choose any encryption method to generate the *DK_u_*. For example, symmetrical key systems such as DES, Triple DES, or AES use diffusion and confusion to block hackers from cracking encryptions through statistical calculations. At present, these password systems remain challenging to crack. Therefore, attackers cannot directly extract meaningful content from encrypted documents in the proposed system.

### 4.3. Collaborative Attack 

Collaborative attack: two or more authorized users collaborate and share superkeys *H_i_* with each other in an attempt to derive a *DK_j_* outside their authorization or other users’ superkeys *H_i_*.

The security class *S_i_* adopted in the proposed method involves partially ordered relationships. When *S_i_* is authorized to access *S_j_*, the following formula *G*(*x*, *y*) can be used:

*G*(*x*, *y*) = *A*_1_(*x*)*B*_1_(*y*) + *A*_2_(*x*)*B*_2_(*y*) + … + *A_n_*(*x*)*B_n_*(*y*) (10)

Therefore, we define a collaborative attack as a situation in which two or more authorized users target another authorized user. Two example scenarios are presented subsequently. In the first scenario, the collaborating attackers are in a partially ordered relationship with their target. In the second scenario, the collaborating attackers are not in a partially ordered relationship with their target.

Scenario 1: the collaborative attackers attempt to collect each other’s privacy parameters superkey *H_i_* and obtain the *DK_u_* of another user in the system that the attackers do not have permission to access. Based on the above Section 3.4.2, the clearance of the collaborating attackers are *S*_3_ = {1, 4} and *S*_4_ = {4} and that of the target is *S*_7_ = {1, 3, 4}. In contrast to *S*_3_ and *S*_4_, *S*_7_ is authorized to access *file*_3_. Therefore, in this example, *S*_3_ and *S*_4_ collaboratively launch an attack to acquire *S*_7_ and *DK*_3_. The data related to *DK*_3_ is hidden in *A*_7_(*x*)*B*_7_(*y*).

$$A_{7}\left(x\right)=\left\{\frac{\left(x-H_{1}\right)\left(x-H_{2}\right)\left(x-H_{3}\right)\left(x-H_{4}\right)\left(x-H_{5}\right)\left(x-H_{6}\right)}{\left(H_{7}-H_{1}\right)\left(H_{7}-H_{2}\right)\left(H_{7}-H_{3}\right)\left(H_{7}-H_{4}\right)\left(H_{7}-H_{5}\right)\left(H_{7}-H_{6}\right)}\right\}\times I_{\left\{H_{1},...,H7\right\}}\left(x\right)$$

$$\begin{array}{c} B_{7}\left(y\right)=\{DK_{1}\times \frac{\left(y-2\right)\left(y-3\right)\left(y-4\right)\left(y-5\right)}{\left(1-2\right)\left(1-3\right)\left(1-4\right)\left(1-5\right)}+DK_{3}\times \frac{\left(y-1\right)\left(y-2\right)\left(y-4\right)\left(y-5\right)}{\left(3-1\right)\left(3-2\right)\left(3-4\right)\left(3-5\right)}\\ +DK_{4}\times \frac{\left(y-1\right)\left(y-2\right)\left(y-3\right)\left(y-5\right)}{\left(4-1\right)\left(4-2\right)\left(4-3\right)\left(4-5\right)}\}\times I_{J_{7}}\left(y\right) \end{array}$$

However, *S*_3_ and *S*_4_ only possess *H*_3_ and *H*_4_, and these superkeys are unable to pass the *A*_7_(*x*) test. Lagrange interpolation calculations using these superkeys yield empty values. Therefore, *A*_7_(*x*)*B*_7_(*y*) = 0 × *B*_7_(*y*) = 0. In this instance, a collaborative attack is similar to an independent attack, and the attackers are not able to obtain additional data.

Scenario 2: the collaborating attackers are not in a partially ordered relationship with their target. The attackers collect each other’s parameters to increase the probability of deriving *DK_u_*. According to Section 4.1, the clearances of the collaborating attackers are *S*_3_ = {1, 4} and *S*_4_ = {4}, and that of the target is *S*_5_ = {5}. As described, no partially ordered relationship exists between *S*_5_ and *S*_3_ or *S*_4_. To obtain *S*_5_ and gain access to *file*_5_, *S*_3_ and *S*_4_ must collaboratively launch an attack on *S*_5_ to obtain *DK*_5_. As in Scenario 1, *S*_3_ and *S*_4_ only possess the superkeys *H*_3_ and *H*_4_, which cannot be used to pass the *A*_5_(*x*) test, and calculations only yield empty values.

Thus, private superkeys *H_i_* cannot be collected to derive a *DK_u_* without authorization, regardless of the number of attackers or whether a partially ordered relationship exists between the attacker(s) and the target.

In addition to *DK_u_*, attackers may also target superkeys *H_i_*. In Scenario 1, the *A*_7_(*x*) results indicate that *S*_3_ and *S*_4_ only possess *H*_3_ and *H*_4_. Thus, these users lack sufficient data to obtain *H*_7_ from the *A*_7_(*x*) results produced through Lagrange interpolation. Therefore, collaborative attacks are ineffective against the proposed system.

### 4.4. Reverse Attack

Reverse attack: an authorized user (attacker) uses a known public formula *G*(*x*, *y*) and his or her private parameters to obtain the superkeys *H_i_^’^* of other users.

Based on Section 3.4.2, users with *S*_6_ and *S*_7_ are generally able to derive *DK*_1_ by applying *G*(*x, y*). *S*_6_ is in a partially ordered relationship with *S*_7_. Specifically, *S*_6_ ≼ *S*_7_, where *S*_6_ = {1} and *S*_7_ = {1, 3, 4}. In this scenario, the user that corresponds with *S*_6_ is the attacker that attempts to use *H*_6_ and *G*(*x, y*) to derive the *H*_7_ of *S*_7_ and then use *S*_7_ to obtain *file*_3_ and *file*_4_.

The proposed method only involves one public formula: *G*(*x*, *y*) = *A*_1_(*x*)*B*_1_(*y*) + … + *A*_6_(*x*)*B*_6_(*y*) + *A*_7_(*x*)*B*_7_(*y*). To use *S*_6_ in the deduction process, point (*H*_6_, 1) is incorporated into the aforementioned polynomials for calculation. Subsequently, *S*_7_ can be used to incorporate points (*H*_7_, 1), (*H*_7_, 3), and (*H*_7_, 4) into the calculations and thereby derive the key allocated by the CA. However, *DK*_3_ and *DK*_4_ of *file*_3_ and *file*_4_ cannot be obtained through incorporating *S*_6_ to point (*H*_6_, 3) or point (*H*_6_, 4).

The user corresponding with *S*_6_ attempts to acquire the *DK*_3_ and *DK*_4_ of *S*_7_. Therefore, the target is *H*_7_ or *DK*_3_ and *DK*_4_ related to *A*_7_(*x*)*B*_7_(*y*)_7_. Because *S*_6_ can be used to incorporate point (*H*_6_, 1) into *G*(*H*_6_, 1) = *DK*_1_, the user corresponding with *S*_6_ may attempt the following calculations: 

*G*(*H*_6_, 1) − *DK*_1_ = 0

⇒*A*_1_(*H*_6_)*B*_1_(1) + … + *A*_6_(*H*_6_)*B*_6_(1) + *A*_7_(*H*_6_)*B*_7_(1) + … + *A_n_*(*H*_6_)*B_n_*(1) − *DK*_1_ = 0

⇒ *c*_0_*d*_0_ + *c*_1_*d*_0_*x* + *c*_0_*d*_1_*y* + *c*_1_*d*_1_*xy* + … + *c*_*n*−1_*d*_*m*−1_*x*^*n*−1^*y*^*m*−1^ − *DK*_1_ = 0

The equation demonstrates that *G*(*x*, *y*) is an (*n* − 1)(*m* − 1)^th^ order polynomial with two unknowns. The attacker cannot decipher the data of *A*_7_(*x*)*B*_7_(*y*) from the polynomials. *G*(*x*, *y*) is extremely simple and does not contain numerous parameters that attackers can manipulate. Even if the attacker gains a portion of *A*_7_(*x*)*B*_7_(*y*), *A*_7_(*x*) and *B*_7_(*y*) are still protected by separate mechanisms.

*H*_7_ data are hidden in *A*_7_(*x*), which is generated through Lagrange interpolation, expressed as follows:

$A_{7}\left(x\right)=\left\{\frac{\left(x-H_{1}\right)\left(x-H_{2}\right)\left(x-H_{3}\right)\left(x-H_{4}\right)\left(x-H_{5}\right)\left(x-H_{6}\right)}{\left(H_{7}-H_{1}\right)\left(H_{7}-H_{2}\right)\left(H_{7}-H_{3}\right)\left(H_{7}-H_{4}\right)\left(H_{7}-H_{5}\right)\left(H_{7}-H_{6}\right)}\right\}\times I_{\left\{H_{1},...,H7\right\}}\left(x\right)$, *A*_7_(x) verifies whether the *H_i_* inputted by the user is present in the verification list approved by the CA. If the user is not approved by the CA, then the *H_i_* is rejected from $I_{\left\{H_{1},...,H_{n}\right\}}\left(x\right)$. If the user uses a superkey other than *H*_7_, Lagrange interpolation calculation yields a value of 0.

*DK*_3_ and *DK*_4_ data are hidden in *B*_7_(y), which is generated through Lagrange interpolation.

$$\begin{array}{c} B_{7}\left(y\right)=\{DK_{1}\times \frac{\left(y-2\right)\left(y-3\right)\left(y-4\right)\left(y-5\right)}{\left(1-2\right)\left(1-3\right)\left(1-4\right)\left(1-5\right)}+DK_{3}\times \frac{\left(y-1\right)\left(y-2\right)\left(y-4\right)\left(y-5\right)}{\left(3-1\right)\left(3-2\right)\left(3-4\right)\left(3-5\right)}\\ +DK_{4}\times \frac{\left(y-1\right)\left(y-2\right)\left(y-3\right)\left(y-5\right)}{\left(4-1\right)\left(4-2\right)\left(4-3\right)\left(4-5\right)}\}\times I_{J_{7}}\left(y\right) \end{array}$$

Users’ file access clearance must be approved by the CA to pass authentication of $I_{J_{i}}\left(y\right)$, where *J_i_* = {*j*: 1 ≤ *j* ≤ *m*}. Otherwise, the function yields an empty value.

Resources outside of individuals’ authorization cannot be retrieved by reversing polynomials. In sum, the proposed method blocks equation attacks.

### 4.5. Proof of Lagrange Interpolation Theorem

In this subsection, we prove the used Lagrange interpolating polynomial is secure so that the above-mentioned malicious attacks, including equation attack, external attack, collaborative attack, and reverse attack are meaningless for our scheme. The proof is shown as follows:

**Theorem 1 (Lagrange interpolation)**: given *t* distinct points (*x_i_*, *y_i_*) of the form (*x_i_*, *f*(*x_i_*)), where *f*(*x*) is a polynomial of degree less that *t*, then *f*(*x*) is determined by:

(11) $$f\left(x\right)=\overset{t}{\underset{i=1}{\sum }}y_{i}\overset{\;}{\underset{\begin{array}{c} 1\leq j\leq t\\ i\neq j \end{array}}{\prod }}\frac{x-x_{i}}{x_{i}-x_{j}}$$

The scheme of Shamir [30] is defined for a secret sϵZ/pZ with *p* prime, by setting *a*_0_ = *s*, and selecting *a*_1_, ..., *a_t_*_−1_ randomly in Z/pZ. The trusted party computes *f*(*i*), where:

(12) $$f\left(x\right)=\overset{t-1}{\underset{k=0}{\sum }}a_{k}x^{k}$$

For all 1 ≤ *i* ≤ *n*. The shares (*i*, *f*(*i*)) are distributed to the *n* distinct parties. Due to the fact that the secret is the constant term *s* = *a*_0_ = *f*(0), the secret is regained from any *t* shares (*i*, *f*(*i*)), for *I* ⊂ {1, ..., *n*} by $s=\overset{\;}{\underset{i\in I}{\sum }}c_{i}f\left(i\right)$, where each $c_{i}=\overset{\;}{\underset{\begin{array}{c} j\in I\\ j\neq i \end{array}}{\prod }}\frac{i}{j-i}$.

**Exercise**: prove the formula for the secret to be accurate by replacing into the formula of Lagrange’s interpolation theorem. 

**Properties**: rhe features of Shamir’s secret sharing scheme are as follows: (1) all hypotheses are under proof, (2) perfec*t* - all information is well-protected by the shares, and (3) ideal - every share is of the same size *p* as the secret. Comparatively, almost all public key cryptosystems depend on some familiar problems (discrete logarithm problems, integer factorization) for hardness so that the safety can be assured.

**Proof of Lagrange interpolation theorem**: suppose *g*(*x*) is the right-hand side of equation (11). For each *x_i_* in we verify directly that *f*(*x_i_*) = *g*(*x_i_*), so as we can get *f*(*x*) – *g*(*x*) is divisible by *x* – *x_i_*. It follows that:

(13) $$\overset{t}{\underset{i=1}{\prod }}\left(x-x_{i}\right)|\left(f\left(x\right)-g\left(x\right)\right)$$

However, because deg(*f*(*x*) − *g*(*x*)) ≤ *t*, the only polynomial of this degree satisfying Equation (13) is *f*(*x*) − *g*(*x*) = 0. □

### 4.6. Problems with Multi-User Access Requests

The proposed digital-data-sharing system is a user-centered structure that integrates all kinds of teaching materials from different users. The collected data are stored in cloud servers to achieve the purpose of digital information integration and resources share and exchange. 

Cloud computing environments show the characteristics of easy expansion and resource share in which it presents several advantages to satisfy the integration, share and exchange of digital materials. In such digital-data-sharing system, the requirements of users to rapidly propose access request and receive permission from cloud service providers should be satisfied.

For this reason, dynamic access schemes need to be established completely to ensure providing instant and entire services of digital data. The key is the services provided by the sharing system being able to support distinct dynamic access demands so as to correspond to the data and user change in cloud computing environments.

The proposed scheme and method are flexible and could deal with all the security management problems of dynamic keys, such as adding a new security class, removing an existing security class, and updating a user authorized. The involved solutions are simple, mainly addition and deduction, that it does not require enormous computation and storage space for parameter update. Regarding the key-deriving formula *G*(*x*, *y*) in Section 3:

$$G\left(x,y\right)=\overset{n}{\underset{i=1}{\sum }}A_{i}\left(x\right)B_{i}\left(y\right)$$

Function *A_i_*(*x*) is related to information verification for verifying the existence of *H_i_* in the legal verification list of CA and the use of personal superkey for verification. Function *B_i_*(*y*) relates to the data verification for verifying the authorization of a user to obtain the decryption key *DK_u_* to further decrypt the encrypted digital materials. 

The dynamic access requirements of sharing system in cloud are considered from two aspects: users and material data. First, users are changeable. Unlike static access model, which could establish all user parameters in the beginning of access scheme, the constant increase or removal of material authors, students, parents, publishers, and various teachers could propose new requests to the user-centered sharing system. User parameters need to be continuous updated to the initial access scheme to correspond to the dynamic users.

Second, material files require appending and revision. The integration of digital data comes from the different users, units, and information sources. In addition to the author, authorized users with requests should be able to update the material records and revise the documents in the sharing system. For this reason, the data of the materials could be appended and removed with dynamic requests after the establishment of access scheme.

In regard to the above considerations, the established formula *G*(*x*, *y*) is nimble and flexible, which could be easily updated and revise the parameters instantaneously. 

### 4.7. Discussion

In this subsection, we discuss the computational overheads and storage required for use of the proposed system. Definitions of notations used in performance evaluation of the proposed scheme are presented in Table 7.

Knuth demonstrated that the process of interpolating at (*n* + 1) points requires (*n*^2^ + *n*)/2 divisions and (*n*^2^ + *n*) subtractions by Newton’s formula, where *n* is the degree of the interpolating polynomial [46].

With regard to the evaluation of the polynomial for the derivation of the successor’s secret parameters, Knuth demonstrated that this scheme requires (2*n −* 1) multiplications and (2*n*) additions in addition to one modular operation performed by applying Horner’s rule.

Therefore, as Table 8 shows, the proposed scheme requires $2nT_{l()}+nT_{mul}$ to create *G*(*x*, *y*) in the process of key generation, where T*_l_*_()_ is the computation time for the interpolating polynomial. As described, the required computations are as follows: *T_l_*_()_ = (2*n −* 1) multiplications + (2*n*) additions + 1 modular operation, $\left(\underset{1\leq i\leq n}{\sum }v_{i}+n\right)T_{l()}+nT_{mul}$. Thus, in total, this process spends $\left(\underset{1\leq i\leq n}{\sum }v_{i}+3n\right)T_{l()}+2nT_{mul}$. In terms of storage, the public parameters *G*(*x*, *y*), *u* in this study require (*m* + 1)|*p*|, and the storage for each security class of a private key *H_i_* is |*p*|.

### 4.8. Comparison

With the advent of the era of cloud computing, the values of access mechanisms lie in their compatibility with various Internet applications as well as their security and efficiency. In this subsection, we compare confidentiality, data integrity, correctness and completeness, time complexity, and whether the key encryption scheme is possessed with other presented schemes. As showed in Table 9, four schemes proposed by Chung et al. [31], Liu et al. [15], Hsiao et al. [47], and ours achieve privacy protection (using notation O to express) due to their owning the key mechanism for encryption data. Specially, Hsiao et al.’s [47] and our schemes also provide access control function and are thus suitable for cloud environments. In respect of time complexity, Chung et al.’s method [31] is based on an elliptic curve cryptosystem, Liu et al.’s scheme [15] is based on the bilinear pairing, and Hsiao et al.’s model [47] is based on the discrete logarithm problem, time taken is exponential time and the time complexity is O(2*^N^*). The proposed scheme is based on the lagrange interpolation polynomial, time complexity is only O(*N*). Due to no key generation and derivation process for the control systems of Trnka and Cerny [40] and A. S. M. Kayes et al. [37], no discussion occurred here. In addition, in both schemes, there is no encryption function for access files. The user who wants to access files can only be determined by the access control, their confidentiality are thus partially achieved (using notation ∆ to express). Finally, all schemes are correctly completed on all process designs, so they can achieve correctness and completeness.

## 5. Conclusions and Future Works

Cloud-based education has been actively promoted in recent years. Amid these efforts, the promotion of digital sharing systems is essential to ensure user and data security. To harness the immense benefits of cloud computing, we developed a cloud-based and learner-centered access control mechanism suitable for multi-user applications. The mechanism resolves the problems of managing numerous users and reduces the complexity of access relationships. It also ensures the confidentiality and integrity of user data stored in the cloud and prevents unauthorized individuals from randomly accessing or modifying digital data. The integrated learning feature of sharing systems prevents repeated investment and development, protects the natural environment, and enhances economic efficiency. Therefore, these systems may be used to facilitate the shift of mobile services to the cloud and stimulate developments in the software industry. 

In the future, we will be strengthening the design for context-awareness to reach the perfect combination of CAAC and RABC with encryption function. In addition to a more stable use for cloud sharing, the scheme will be going further to a wider application in key managements, and access control for the area of Big Data, the Internet of Things, and AI that emphasize dynamic authentication, e.g., voice-command access control, biometric access control, intelligence-learning access control and key-configuration mechanism.

## Figures and Tables

**Table 1:** Symbol definitions and parameters.

Symbol	Definition	Function
*S_i_*	Security class, *S_i_* = {*u*: *u* is the file id that *S_i_* is authorized to access}, for *i* = 1, 2, ..., *n*	To distinguish the user’s security class
*H_i_*	Superkey *H_i_*, for *i* = 1, 2, ..., *n*	To obtain the key for accessing *file_u_*
*DK_u_*	Decryption key, for *u* = 1, 2, ..., *m*	To decrypt the *file_u_* key
*file_u_*	*File_u_*, for *u* = 1, 2, ..., *m*	Represents the file encrypted with *DK_u_*
$I_{\left\{H_{1},...,H_{n}\right\}}\left(x\right)$	The indicate function of set {*H*_1_, *H*_2_, ..., *H_n_*}	To determine whether *H_i_* is present in the authentication list approved by the central authority (CA)
*J_i_*	*J_i_* = {*u*: 1 ≤ *u* ≤ *m*, *u* is the file id that *S_i_* is authorized to access}	User-authorized file set
$I_{J_{i}}\left(x\right)$	The indicate function of set *J_i_*	To determine whether the user has authorized the file set

**Table 2:** Sets of security class and superkey.

*S*_1_	*S*_2_	…	*S_i_*	…	*S_n_*	
*H*_1_	*H*_2_	…	*H_i_*	…	*H_n_*	←secret and distinct

**Table 3:** Decryption keys for corresponding encrypted files.

*file*_1_	*file*_2_	…	*file_u_*	…	*file_m_*	
1	2	…	*u*	…	*m*	file id, public
*DK*_1_	*DK*_2_	…	*DK_u_*	…	*DK_m_*	decryption keys, secret and distinct

**Table 4:** The results after adding a new security class.

	*file*_1_(*DK*_1_) Junior High School Year 1 English	*file*_2_(*DK*_2_) Junior High School Year 2 Math.	*file*_3_(*DK*_3_) Senior High School Year 2 Physics	*file*_4_(*DK*_4_) Senior High School Year 3 Chemistry	*file*_5_(*DK*_5_) University Year 1 Chinese
***S*_1_(*H*_1_):** Teaching material author	1	1	1	1	1
***S*_2_(*H*_2_):** Publisher	1	1	1	1	0
***S*_3_(*H*_3_):** Teacher	1	0	0	1	0
***S*_4_(*H*_4_):** Student	0	0	0	1	0
***S*_5_(*H*_5_):** Cram school operator	0	0	0	0	1
***S*_6_(*H*_6_):** Student parents	1	0	0	0	0
***S*_7_(*H*_7_):** Downstream bookstore	1	0	1	1	0

**Table 5:** The resulting after revoking the existing current security class.

	*file*_1_(*DK*_1_) Junior High School Year 1 English	*file*_2_(*DK*_2_) Junior High School Year 2 Math.	*file*_3_(*DK*_3_) Senior High School Year 2 Physics	*file*_4_(*DK*_4_) Senior High School Year 3 Chemistry	*file*_5_(*DK*_5_) University Year 1 Chinese
***S*_1_(*H*_1_):** Teaching material author	1	1	1	1	1
***S*_2_(*H*_2_):** Publisher	1	1	1	1	0
***S*_3_(*H*_3_):** Teacher	1	0	0	1	0
***S*_4_(*H*_4_):** Student	0	0	0	1	0
***S*_5_(*H*_5_):** Cram school operator	0	0	0	0	1
***S*_6_(*H*_6_):** Student parents	1	0	0	0	0

**Table 6:** The resulting after updating of a user authorized.

	*file*_1_(*DK*_1_) Junior High School Year 1 English	*file*_2_(*DK*_2_) Junior High School Year 2 Math	*file*_3_(*DK*_3_) Senior High School Year 2 Physics	*file*_4_(*DK*_4_) Senior High School Year 3 Chemistry	*file*_5_(*DK*_5_) University Year 1 Chinese
***S*_1_(*H*_1_):** Teaching material author	1	1	1	1	1
***S*_2_(*H*_2_):** Publisher	1	1	1	1	0
***S*_3_(*H*_3_):** Teacher	1	0	0	1	0
***S*_4_(*H*_4_):** Student	0	1	0	0	0
***S*_5_(*H*_5_):** Cram school operator	0	0	0	0	1
***S*_6_(*H*_6_):** Student parents	1	0	0	0	0
***S*_7_(*H*_7_):** Downstream bookstore	1	1	1	1	1

**Table 7:** Notation table.

Definition	Notation
*n*	Number of security classes
*m*	Number of files
*ν*_i_	Degree of the polynomial *f*(x) (the system involves *N* security classes, each with *v_i_* predecessors)
|*p*|	Bit-length of an integer *p*
*T_l()_*	Time required to calculate an interpolating polynomial
*T_mul_*	Time required for a multiplication computation

**Table 8:** Analysis of computation complexity.

	Key Generation/Derivation	Storage of Public Parameters	Storage of Private Keys for Each Security Class
**Proposed scheme**	$\left(\underset{1\leq i\leq n}{\sum }v_{i}+3n\right)T_{l()}+2nT_{mul}$	(m+1)|*p*|	|*p*|

**Table 9:** Comparison with security requirements.

	(1)	(2)	(3)	(4)	(5)
Chung et al. (2008) [31]	O	Cryptography	O	O(2*^N^*)	Yes
Liu et al. (2013) [15]	O	Cryptography	O	O(*2^N^*)	Yes
Trnka and Cerny (2016) [40]	∆	Access control	O	-	No
Hsiao et al. (2018) [47]	O	Access control and cryptography	O	O(*2^N^*)	Yes
A. S. M. Kayes et al. (2019) [37]	∆	Access control	O	-	No
Our proposal (2019)	O	Access control and cryptography	O	O(*N*)	Yes
(1) Confidentiality; (2) data integrity; (3) correctness and completeness; (4) complexity; (5) privacy protection.					

## References

[1] Fosnot C.T., Perry R.S. (2005). Constructivism: A Psychological Theory of Learning. Constructivism: Theory, Perspectives, and Practice.

[2] Woolley D.R. (1994). PLATO: The emergence of on-line community. Comput.-Mediated Commun. Mag..

[3] Pivec M., Dziabenko O., Schinnerl I. Aspects of game-based learning. Proceedings of the 3rd International Conference on Knowledge Management.

[4] Ebner M., Böckle M., Schön M. Game Based Learning in Secondary Education: Geographical Knowledgeof Austria. Proceedings of the 2011 World Conference on Educational Multimedia, Hypermediaand Telecommunications.

[5] Moschini E. (2006). Designing for the smart player: Usability design and user-centred design in game-based learning. Digit. Creat..

[6] Prensky M. (2003). Digital game-based learning. Comput. Entertain. (CIE).

[7] Mell P., Grance T. (2011). The NIST Definition of Cloud Computing.

[8] Brunette G., Mogull R. (2017). Security Guidance for Critical Areas of Focus in Cloud Computing V4.0.

[9] Gens F. (2009). New IDC It Cloud Services Survey: Top Benefits and Challenges. http://blogs.idc.com/ie/?p=730.

[10] Gai K., Qiu M. (2018). Blend Arithmetic Operations on Tensor-Based Fully Homomorphic Encryption over Real Numbers. IEEE Trans. Ind. Inform..

[11] Gai K., Qiu M., Ming Z., Zhao H., Qiu L. (2017). Spoofing-Jamming Attack Strategy Using Optimal Power Distributions in Wireless Smart Grid Networks. IEEE Trans. Smart Grid.

[12] Carminati B., Colombo P., Ferrari E., Sagirlar G. Enhancing User Control on Personal Data Usage in Internet of Things Ecosystems. Proceedings of the 2016 IEEE International Conference on Services Computing (SCC).

[13] Sandhu R.S., Coyne E.J., Feinstein H.L., Youman C.E. (1996). Role-Based Access Control Models. IEEE Comput..

[14] Li M., Yu S., Ren K., Lou W. Securing Personal Health Records in Cloud Computing: Patient-centric and Fine-grained Data Access Control in Multi-owner Settings. Proceedings of the International Conference on Security and Privacy in Communication Networks.

[15] Liu C.-H., Lin F.-Q., Chiang D.-L., Chen T.-L., Chen C.-S., Lin H.-Y., Chung Y.-F., Chen T.-S. Secure PHR Access Control Scheme for Healthcare Application Clouds. Proceedings of the 2013 42nd International Conference on Parallel Processing.

[16] Saunders G., Hitchens M., Varadharajan V. (2001). Role-Based Access Control and the Access Control Matrix. ACM SIGOPS Oper. Syst. Rev..

[17] Coulouris G., Dollimore J., Roberts M. Role and Task-Based Access Control in the PerDiS Groupware Platform. Proceedings of the 3rd ACM Workshop on Role-Based Access.

[18] Joshi J.B.D., Bertino E., Latif U., Ghafoor A. (2005). A Generalized Temporal Role-Based Access Control Model. IEEE Trans. Knowl. Data Eng..

[19] Ott A., Fischer-Hübner S. (2004). The Rule Set Based Access Control (RSBAC) Framework for Linux. http://www.rsbac.org/documentation/.

[20] Hansen F., Oleshchuk V. SRBAC: A Spatial Role-Based Access Control Model for Mobile Systems. Proceedings of the 7th Nordic Workshop on Secure IT Systems.

[21] Park J.S., Costello K.P., Neven T.M., Diosomito J.A. A Composite RBAC Approach for Large, Complex Organizations. Proceedings of the 9th ACM Symposium on Access Control Models and Technologies.

[22] Wang H., Cao J., Zhang Y. (2005). A Flexible Payment Scheme and Its Role-Based Access Control. IEEE Trans. Knowl. Data Eng..

[23] Sandhu R.S., Munawer Q. The RRA97 Model for Role-Based Administration of Role Hierarchies. Proceedings of the 14th Annual Computer Security Applications Conference.

[24] Sandhu R.S., Samarati P. (1994). Access control: Principle and practice. IEEE Commun. Mag..

[25] Osborn S.L., Sandhu R.S., Munawer Q. (2000). Configuring role-based access control to enforce mandatory and discretionary access control policies. ACM Trans. Inf. Syst. Secur..

[26] Ferraiolo D.F., Sandhu R.S., Gavrila S.I., Kuhn D.R. (2001). Ramaswamy Chandramouli: Proposed NIST standard for role-based access control. ACM Trans. Inf. Syst. Secur..

[27] Chen T.-S., Chung Y.-F. (2002). Hierarchical access control based on Chinese Remainder Theorem and symmetric algorithm. Comput. Secur..

[28] Chen T.-S., Chung Y.-F., Tian C.-S. A Novel Key Management Scheme for Dynamic Access Control in a User Hierarchy. Proceedings of the COMPSAC 2004.

[29] Pan J.-Y., Chen T.-L., Chen T.-S. A Novel Key Management and Access Control Scheme for Mobile Agent. Proceedings of the 2006 International Conference on Intelligent Computing.

[30] Stallings W. (2016). Cryptography and Network Security: Principles and Practice.

[31] Chung Y.-F., Lee H.-H., Lai F., Chen T.-S. (2008). Access control in user hierarchy based on elliptic curve cryptosystem. Inf. Sci..

[32] Huang K.-H., Chung Y.-F., Liu C.-H., Lai F., Chen T.-S. (2009). Efficient migration for mobile computing in distributed networks. Comput. Stand. Interfaces.

[33] Liu C.-H., Chung Y.-F., Chen T.-S., Wang S.-D. Access Control and Key Management Scheme Based on Bilinear Pairings over Elliptic Curves for Mobile Agent. Proceedings of the 2009 Third International Conference on Multimedia and Ubiquitous Engineering.

[34] Liu C.-H., Chung Y.-F., Chen T.-S., Wang S.-D. (2012). Mobile Agent Application and Integration in Electronic Anamnesis System. J. Med. Syst..

[35] Chen T.-S., Liu C.-H., Chen T.-L., Chen C.-S., Bau J.-G., Lin T.-C. (2012). Secure Dynamic Access Control Scheme of PHR in Cloud Computing. J. Med. Syst..

[36] Kayes A.S.M., Rahayu W., Dillon T., Chang E., Han J. (2019). Context-aware access control with imprecise context characterization for cloud-based data resources. Future Gener. Comput. Syst..

[37] Kayes A.S.M., Han J., Rahayu W., Dillon T., Islam M.S., Colman A. (2019). A Policy Model and Framework for Context-Aware Access Control to Information Resources. Comput. J..

[38] Schefer-Wenzl S., Strembeck M. (2013). Modelling context-aware RBAC models for mobile business processes. Int. J. Wirel. Mob. Comput..

[39] Hosseinzadeh S., Virtanen S., Rodríguez N.D., Lilius J. A semantic security framework and context-aware role-based access control ontology for smart spaces. Proceedings of the International Workshop on Semantic Big Data.

[40] Trnka M., Cerny T. On security level usage in context-aware role-based access control. Proceedings of the 31st Annual ACM Symposium on Applied Computing.

[41] Colombo P., Ferrari E. Towards Virtual Private NoSQL datastores. Proceedings of the 2016 IEEE 32nd International Conference on Data Engineering (ICDE).

[42] Colombo P., Ferrari E. (2017). Enhancing NoSQL datastores with fine-grained context-aware access control: A preliminary study on MongoDB. Int. J. Cloud Comput..

[43] Kayes A.S.M., Han J., Colman A. An ontology-based approach to context-aware access control for software services. Proceedings of the International Conference on Web Information Systems Engineering.

[44] Kayes A.S.M., Han J., Colman A., Islam M.S. RelBOSS: A relationship-aware access control framework for software services. Proceedings of the 2014 OTM Confederated International Conferences “On the Move to Meaningful Internet Systems”.

[45] Kayes A.S.M., Han J., Colman A. PO-SAAC: A purpose-oriented situation-aware access control framework for software services. Proceedings of the 2014 International Conference on Advanced Information Systems Engineering.

[46] Szidarovszky F., Yakowitz S. (1978). Principles and Procedures of Numerical Analysis.

[47] Hsiao T.C., Wu Z.Y., Chen T.L., Chung Y.F., Chen T.S. (2018). A hierarchical access control scheme based on Lagrange interpolation for mobile agents. Int. J. Distrib. Sens. Netw..

